# Urine biomarkers individually and as a consensus model show high sensitivity and specificity for detecting UTIs

**DOI:** 10.1186/s12879-024-09044-2

**Published:** 2024-01-31

**Authors:** Marzieh Akhlaghpour, Emery Haley, Laura Parnell, Natalie Luke, Mohit Mathur, Richard A. Festa, Michael Percaccio, Jesus Magallon, Mariana Remedios-Chan, Alain Rosas, Jimin Wang, Yan Jiang, Lori Anderson, David Baunoch

**Affiliations:** 1 Department of Research and Development, Pathnostics, 15545 Sand Canyon Suite 100, Irvine, CA 92618 USA; 2 Department of Clinical Research, Pathnostics, 15545 Sand Canyon Suite 100, Irvine, CA 92618 USA; 3 Department of Scientific Writing, Precision Consulting, 6522 Harbor Mist, Missouri City, TX 77459 USA; 4 Department of Medical Affairs, Pathnostics, 15545 Sand Canyon Suite 100, Irvine, CA 92618 USA; 5 Department of Statistical Analysis, Stat4Ward, 2 Edgemoor Lane, Pittsburgh, PA 15238 USA; 6 Department of Writing, L. Anderson Diagnostic Market Access Consulting, 2755 Eagle Street, San Diego, CA 92103 USA

**Keywords:** Urinary tract infection (UTI), Diagnostic testing, Urinary biomarkers, Neutrophil gelatinase-associated lipocalin (NGAL), Interleukin 8 (IL-8), Interleukin 1β (IL-1β)

## Abstract

**Background:**

Current diagnoses of urinary tract infection (UTI) by standard urine culture (SUC) has significant limitations in sensitivity, especially for fastidious organisms, and the ability to identify organisms in polymicrobial infections. The significant rate of both SUC “negative” or “mixed flora/contamination” results in UTI cases and the high prevalence of asymptomatic bacteriuria indicate the need for an accurate diagnostic test to help identify true UTI cases. This study aimed to determine if infection-associated urinary biomarkers can differentiate definitive UTI cases from non-UTI controls.

**Methods:**

Midstream clean-catch voided urine samples were collected from asymptomatic volunteers and symptomatic subjects ≥ 60 years old diagnosed with a UTI in a urology specialty setting. Microbial identification and density were assessed using a multiplex PCR/pooled antibiotic susceptibility test (M-PCR/P-AST) and SUC. Three biomarkers [neutrophil gelatinase-associated lipocalin (NGAL), and Interleukins 8 and 1β (IL-8, and IL-1β)] were also measured via enzyme-linked immunosorbent assay (ELISA). Definitive UTI cases were defined as symptomatic subjects with a UTI diagnosis and positive microorganism detection by SUC and M-PCR, while definitive non-UTI cases were defined as asymptomatic volunteers.

**Results:**

We observed a strong positive correlation (R^2^ > 0.90; *p* < 0.0001) between microbial density and the biomarkers NGAL, IL-8, and IL-1β for symptomatic subjects. Biomarker consensus criteria of two or more positive biomarkers had sensitivity 84.0%, specificity 91.2%, positive predictive value 93.7%, negative predictive value 78.8%, accuracy 86.9%, positive likelihood ratio of 9.58, and negative likelihood ratio of 0.17 in differentiating definitive UTI from non-UTI cases, regardless of non-zero microbial density. NGAL, IL-8, and IL-1β showed a significant elevation in symptomatic cases with positive microbe identification compared to asymptomatic cases with or without microbe identification. Biomarker consensus exhibited high accuracy in distinguishing UTI from non-UTI cases.

**Conclusion:**

We demonstrated that positive infection-associated urinary biomarkers NGAL, IL-8, and IL-1β, in symptomatic subjects with positive SUC and/or M-PCR results was associated with definitive UTI cases. A consensus criterion with ≥ 2 of the biomarkers meeting the positivity thresholds showed a good balance of sensitivity (84.0%), specificity (91.2%), and accuracy (86.9%). Therefore, this biomarker consensus is an excellent supportive diagnostic tool for resolving the presence of active UTI, particularly if SUC and M-PCR results disagree.

**Supplementary Information:**

The online version contains supplementary material available at 10.1186/s12879-024-09044-2.

## Background

The use of standard urine culture (SUC) to identify classical uropathogens in urinary tract infection (UTI) has been standard practice for several decades, but has several limitations [1]. One such limitation is that SUC uses specific media and conditions that result in cultivating easy-to-grow microbes like *Escherichia coli (E. coli)* yet poorly grows non-*E. coli* pathogens which have been reported as important emerging uropathogens [2–4]. ​​​ Recent studies have increased awareness of many additional clinically relevant microbial species, such as gram-positive organisms, fastidious microbes, and fungi, which can contribute to urinary microbiome dysbiosis in symptomatic subjects [5]. Additionally, studies using more sensitive culture techniques, such as enhanced-quantitative urine culture (EQUC), and culture-free methods such as gene sequencing and MALDI-TOF have also led to the discovery of the uromicrobiome, which is present even in asymptomatic individuals [2–4]. ​​​.

The limitations of SUC, the presence of a urinary microbiome, and the high prevalence of asymptomatic bacteriuria [6–10] underscores the need to develop diagnostic tests that can identify the presence of urinary tract inflammation in UTI symptomatic patients with high sensitivity and specificity. First, these tests will help identify patients with false negative SUC results who are still likely to have a UTI and need appropriate therapy. Second, while the identification of uropathogens with more sensitive tests such as multiplex polymerase chain reaction (M-PCR) is a strong indicator of infection, there remain questions about whether microbes detected using these tests are associated with a UTI and cause inflammation of the urinary tract. Accurate tests that identify true UTI patients would also be important in pediatric cases where symptom elucidation can be problematic or in cognitively impaired patients. For example, in the long-term care setting, there are high rates of both asymptomatic bacteriuria (up to 50%) [9] and cognitive impairment.

With that in mind, there have been hundreds of studies looking at biomarkers as a potential tool for the identification of UTIs [11]. The innate immune system in the urinary tract consists of both resident and recruited cells expressing a variety of pattern recognition receptors that detect pathogens early and rapidly trigger a pro-inflammatory immune response to aid in bacterial clearance until the microbial threat is resolved [12, 13]. ​ Soluble infection-associated biomarkers can be detected in urine, and studies have demonstrated the association of these urinary biomarkers with the presence of a clinically diagnosed UTI.​ [11, 14, 15] Using such biomarkers, individually or in combination, provides strong evidence of immune response to uropathogens in the urinary tract at the time of urine collection. In an unpublished pilot study (*n* = 100), we evaluated five candidate urine markers [neutrophil gelatinase-associated lipocalin (NGAL), interleukins 8, 6, and 1β (IL-8, IL-6, and IL1-β), and matrix metalloproteinase 9 (MMP-9)] selected based on literature [17–28], and found that three showed a promising correlation with uropathogen detection by M-PCR and SUC in patients symptomatic for UTI: NGAL and IL-8 had good sensitivity and specificity while IL-1β had very high sensitivity (Supplemental Table S2).

Neutrophil gelatinase-associated lipocalin (NGAL), also known as lipocalin-2, is a bacteriostatic agent secreted by uroepithelial cells. Increased urine NGAL levels has been found in rat models of UTI and women with UTIs [19–21]. Interleukin 8 (IL-8), also known as chemokine ligand 8 (CXCL8) [17, 22–26], and IL-1β [22, 27, 28] are both pro-inflammatory cytokines secreted by resident and recruited immune cells. In this study, we aimed to validate whether these three biomarkers can differentiate “definitive UTI” defined as subjects who were symptomatic, with a diagnosis of UTI in a urology specialty setting, and who had positive microbe detection from “definitive non-UTI” defined as asymptomatic subjects either with microbes detected in the urine (asymptomatic bacteriuria) or without.

## Methods

### Study design and participants

Results from biomarker analyses, M-PCR/P-AST tests, and standard urine culture (SUC) included in this analysis were obtained from urine samples from two clinical studies: One was a prospective observation study (WCG IRB 20230847) that enrolled subjects 60 years of age or older who were asymptomatic for UTI. Subjects were recruited from the community (at theaters, sporting events, social gatherings, etc.) and provided written informed consent prior to enrollment. Subjects who were pregnant, taking antibiotics for a UTI, or who have cancer of the urinary tract were excluded. A total of 228 asymptomatic subjects from two states were enrolled in the study between 2/28/2023 and 3/22/2023. All subjects in the study completed the validated American English Acute Cystitis Symptom Score (ACSS) baseline questionnaire and a short medical history (Supplemental Table S19) and provided a midstream voided urine specimen [32]. Symptom status was determined using the US Food and Drug Administration (FDA) symptom scores on the validated American English Acute Cystitis Symptom Score (ACSS) Questionnaire, asking patients to evaluate four typical UTI symptoms: urinary frequency, urinary urgency, dysuria, and suprapubic pain, as well as visible blood in the urine, according to each one’s severity (scoring 0–3): no (0), mild (1), moderate (2), severe (3). Asymptomatic cases were defined as having four FDA symptom scores adding up to < 4, none of the four symptom scores being > 1, and the absence of visible blood in the urine.

The other was a biorepository study from which the symptomatic cohort samples were obtained. Urine samples from patients 60 years of age or older who presented to outpatient urology clinics in 39 states with symptom(s) and ICD-10-CM codes consistent with UTI were collected, de-identified, and stored into the biorepository bank with 583 urine samples accrued in the bank between 01/17/2023 and 04/24/2023. Each de-identified urine sample was assigned a repository label associated with a record of the subject’s age, sex, and ICD-10-CM code(s) and stored in a biorepository for evaluation at Pathnostics’ clinical laboratory. The WCG IRB deemed the biorepository-obtained specimens exempt from review under 45 CFR § 46.104(d)(4), as data from the study was collected via a deidentified database and used in a manner that the identity of the subject cannot be readily ascertained directly or through identifiers linked to the subjects, and that the investigator would not contact or re-identify the subjects.

Urine specimens from both studies were collected via the midstream clean-catch/voided method. Results from biomarker analyses, M-PCR/P-AST, and SUC performed side by side from the urine samples from these 228 asymptomatic subjects and 583 symptomatic subjects were analyzed to investigate if infection-associated urine biomarkers can differentiate definitive UTIs from non-UTI controls.

### The Guidance^®^ UTI M-PCR/P-AST assay (Pathnostics in Irvine, CA)

The test includes susceptibility testing for 19 antibiotics, semi-quantification of 27 distinct uropathogenic species and three bacterial groups, as well as identification of 32 antibiotic-resistance genes and the ESBL phenotype. The test was performed as described previously: the first step involves DNA extraction from the subject’s urine sample using King Fisher/MagMAX™ automated DNA extraction instrument and the MagMAX™ DNA Multi-Sample Ultra Kit (Thermo Fisher Scientific, Carlsbad, CA) per the manufacturer’s instructions. Extracted DNA was mixed with a universal PCR master mix and amplified using TaqMan technology in a Life Technologies 12 K Flex 112-format OpenArray System (Thermo Fisher Scientific, Wilmington, NC). Probes and primers were used to detect 23 bacterial species and 3 bacterial groups, fastidious and non-fastidious, and four yeast species [16–18] listed below:

Classical uropathogens: *Candida albicans, Candida glabrata, Candida parapsilosis, Citrobacter freundii, Citrobacter koseri, Enterococcus faecalis, Enterococcus faecium, Escherichia coli, Klebsiella oxytoca, Klebsiella pneumoniae, Morganella morganii, Pantoea agglomerans, Proteus mirabilis, Providencia stuartii, Pseudomonas aeruginosa, Serratia marcescens, Staphylococcus aureus, Streptococcus agalactiae*, and *Enterobacter group [including Klebsiella aerogenes* (formally known as *Enterobacter aerogenes)* and *Enterobacter cloacae]*.

Emerging uropathogens: *Acinetobacter baumannii, Actinotignum schaalii, Aerococcus urinae, Alloscardovia omnicolens, Candida auris, Corynebacterium riegelii, Gardnerella vaginalis, Mycoplasma hominis, Ureaplasma urealyticum*, coagulase-negative *staphylococci* group (CoNS) (including *Staphylococcus epidermidis, Staphylococcus haemolyticus, Staphylococcus lugdunesis, and Staphylococcus saprophyticus)*, and Viridans group *streptococci* (VGS) (including *Streptococcus anginosus, Streptococcus oralis*, and *Streptococcus pasteuranus*).

Results of the P-AST portion of the test, a pooled antibiotic susceptibility assay which accounts for bacterial interactions, were not included in this analysis.

### Standard urine culture (SUC)

The SUC method was performed as previously described [16]. ​ Briefly, urine was vortexed, and a sterile plastic loop (1 μL) was used to inoculate blood agar plates. A sterile plastic loop (1 μL) was used also to inoculate colistin and nalidixic acid agar/MacConkey agar (CNA/MAC) plates, one loop-full of urine on the CNA side of the plate and another full loop-full on the MAC side of the plate. All plates were incubated at 35^o^ C in 5% CO_2_ for ≥ 18 h and then examined for evidence of growth. Per standard operating procedures plates with < 10,000 CFU/mL were reported as normal urogenital flora [19]. For plates with growth (≥ 10,000 CFU/mL), the quantity and morphology of each organism were recorded. The maximum readable colony count using the 1 μL loop is > 100,000 CFU/mL. Colony counts were performed on blood agar plates. Species identification and colony counts were performed on CNA/MAC plates. Pathogen identification was confirmed with the VITEK 2 Compact System (bioMerieux, Durham, NC).

### Enzyme-linked immunosorbent assay (ELISA)

Urine levels of NGAL, IL-8, and IL-1β were analyzed according to the manufacturer’s instructions, using ELISA kits from R&D Systems/Bio-Techne (Minneapolis, MN), including human Lipocalin-2 / NGAL Quantikine ELISA Kit (Catalog number SLCN20), human IL-8 / CXCL8 Quantikine ELISA Kit (Catalog number S8000C), and human IL-1β / IL-1F2 Quantikine ELISA kit (Catalog number SLB50). OD readings at 450 and 540 nm, respectively, were measured on an Infinite M Nano + microplate reader (TECAN, Switzerland).

### Statistical analysis

Participant demographics and ICD-10-CM code breakdown were described by summary statistics (e.g., mean and standard deviation (SD) for continuous variables such as age, count, and percentage for categorical variables such as sex and ICD-10-CM code). To evaluate the ability of the biomarkers to differentiate UTI from non-UTI conditions such as asymptomatic bacteriuria, we defined “Definitive UTI cases” and “Definitive non-UTI cases.” Definitive UTI cases were defined using the current standard of care diagnostic criteria of symptoms/clinical presentation by urology/urogynecology specialists combined with the presence of microorganisms in the urine above a certain density threshold and being positive by *both* SUC and M-PCR (“Both Detected”). Definitive non-UTI cases were defined as asymptomatic subjects regardless of the presence of detectable microbes in the urine.

After conducting a comprehensive power analysis, our results demonstrate that with a sample size of 351 cases of definitive Urinary Tract Infection (UTI) and 228 cases of definitive non-UTI, we can reliably detect effect sizes as small as 0.24 (Cohen’s d). This analysis was performed considering an 80% statistical power and a significance level of 0.05. This indicates a solid capability to identify subtle differences between the two groups, with a minimal risk of false positives.

Although 100,000 CFUs/mL by SUC is typically considered diagnostically significant in the US, clinical reviews and guidelines, as well as our data suggest a microbial density threshold of 10,000 cells/mL or CFUs/mL is more clinically relevant [20–27]. Thus, we performed analyses using both microbial density thresholds of positivity: Criterion 1 (10,000 cells/mL by M-PCR or CFUs/mL by SUC) and Criterion 2 (100,000 cells/mL by M-PCR or CFUs/mL by SUC). Results using criterion 1 are presented in the main manuscript, while results using criterion 2 are included in the supplemental section.

### Criterion 1 definitions

#### Definitive UTI cases

Symptomatic cases where M-PCR detected bacterial counts of ≥ 10,000 or yeast counts > 0 cells/mL and SUC detected bacterial counts of ≥ 10,000 or yeast counts > 0 CFUs/mL.

#### Definitive non-UTI cases

All asymptomatic cases regardless of microbe identification and density.

### Criterion 2 definitions (Supplemental Data)

#### Definitive UTI cases

Symptomatic cases where M-PCR detected bacterial counts of ≥ 100,000 or yeast counts > 0 cells/mL and SUC detected bacterial counts of ≥ 100,000 or yeast counts > 0 CFUs/mL.

#### Asymptomatic cohort

All asymptomatic cases regardless of microbe identification and density.

Biomarker thresholds previously reported in literature were used to determine positive and negative results for the biomarkers (Table 1). Consensus biomarker positivity was defined as ≥ 2 of the 3 biomarkers measuring at or above their respective cutoff values. A probit regression was fitted and plotted to describe the relationship between the density of organisms detected and the positivity (proportion of samples from symptomatic and asymptomatic cohorts with biomarker levels above the threshold) for each biomarker. Statistical analysis between sensitivity of different individual or combinations of biomarkers used a Proportion Z-test. Statistical difference was defined as *p* < 0.05. The confidence intervals of the biomarker clinical performance characteristics (sensitivity, specificity, positive predictive value, negative predictive value, accuracy, positive likelihood ratio, and negative likelihood ratio) were calculated using the exact method.

**Table 1:** Biomarker Positivity Cutoffs

Biomarker	Cutoff
NGAL	≥ 38.0 ng/mL
IL-8	≥ 20.6 pg/mL
IL-1β	≥ 12.4 pg/mL

All the statistical analyses were performed using R 4.2.2 (https://www.r-project.org/).

## Results

### Demographics

A total of 811 unique subjects’ urine specimens, 583 from the symptomatic cohort and 228 from the asymptomatic cohort were analyzed. The subjects in the symptomatic cohort trended slightly older [mean 76.6, median 76.3, range 60.0–99.0 years] than subjects in the asymptomatic cohort [mean 68.8, median 67.5 years, range 60.0–94.0]. There were also a greater proportion of females in the symptomatic cohort (68.3%, *n* = 398) than in the asymptomatic cohort 55.7% (*n* = 127). Most symptomatic subjects had an ICD-10-CM code (https://www.icd10data.com) of N39.0 for Urinary Tract Infection, site not specified (81.8%, *n* = 534) (Supplemental Table S1). The asymptomatic cohort specimens were collected from volunteers from the general population and therefore, had no ICD-10-CM codes.

### Correlation relationships between biomarker percent positivity and microbial density by M-PCR

**Fig. 1:**
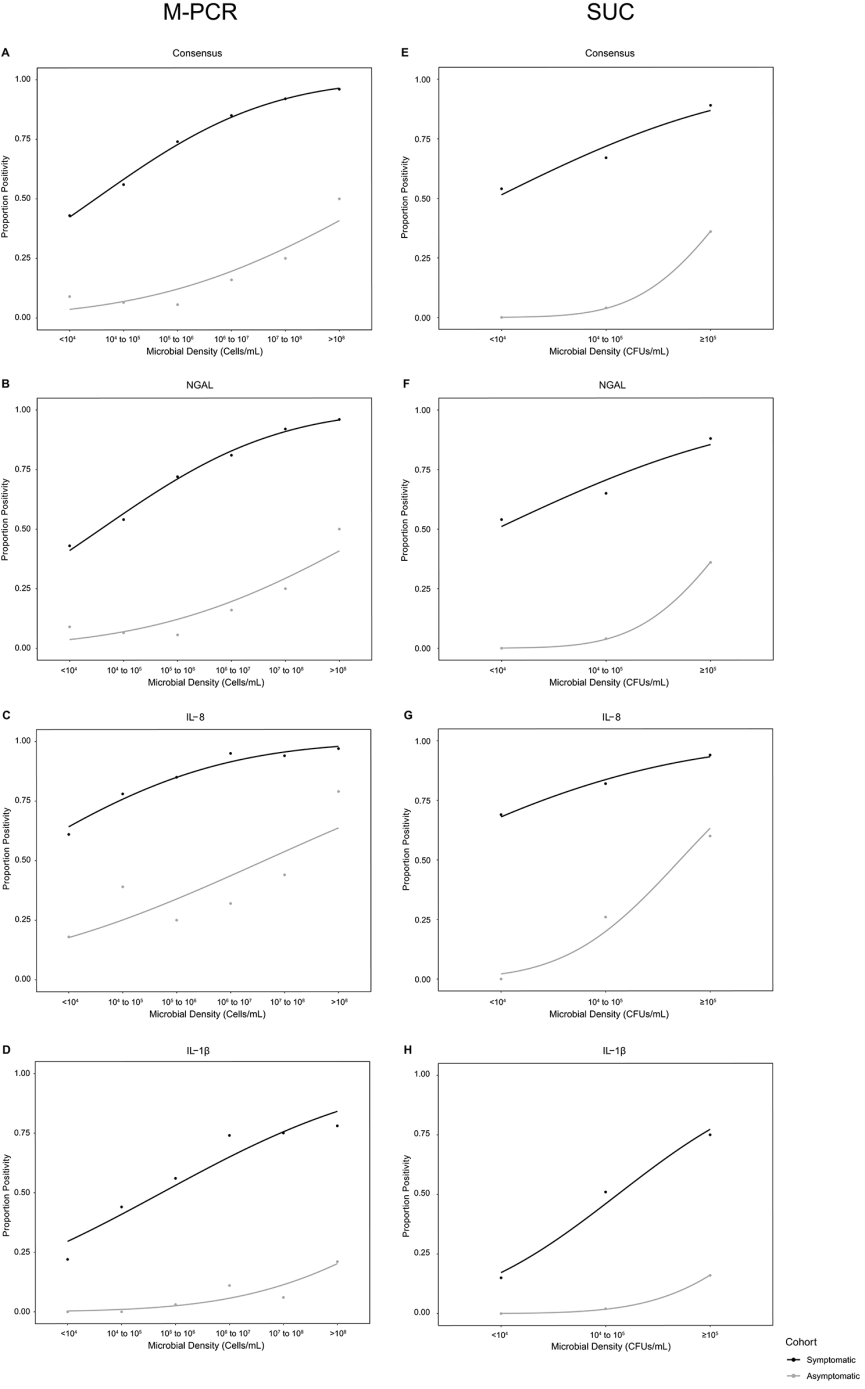
Positive Correlation Between Microbial Density by M-PCR and Biomarker Positivity. The probit regression lines demonstrate a significant positive correlation between urine microbial density and biomarker consensus (**A**, **E**), NGAL (**B**, **F**), IL-8 (**C**, **G**), and IL-1β (**D**, **H**) positivity in both symptomatic (black), and asymptomatic (grey) subjects. Each data point indicates the proportion of biomarker positivity (x-axis) for urine specimens at each of the semi-quantitatively reported microbial densities in cells/mL (≤ 10^4^ 10^4^ to 10^5^, 10^5^ to 10^6^, 10^6^ to 10^7^, 10^7^ to 10^8^, and ≥ 10^8^ for M-PCR or ≤ 10^4^, 10^4^ to 10^5^, and ≥ 10^5^ for SUC) presented along the y-axis. A probit regression analysis line is shown connecting the data points

First, we examined the correlation between biomarker positivity and microbial density by M-PCR in both urine samples from symptomatic and asymptomatic subjects (Fig. 1). Each probit regression for symptomatic subjects had an R^2^ > 0.90 and a *p*-value of < 0.0001 for all biomarkers in the symptomatic cohort. For the asymptomatic cohort, the probit regressions had R^2^ values < 0.90 for M-PCR microbial densities, but > 0.96 for SUC microbial densities and all *p*-values were < 0.05 for all biomarkers, indicating that the correlation between microbial density and biomarker positivity is statistically significant.

Although the symptomatic and asymptomatic cohorts both exhibited a strong positive correlation between biomarker positivity and microbial density, the biomarker proportion positivity was considerably higher across all microbial densities in symptomatic subjects relative to asymptomatic subjects (Fig. 1A– H).

### Comparison of biomarker levels between asymptomatic and symptomatic cohorts

**Fig. 2:**
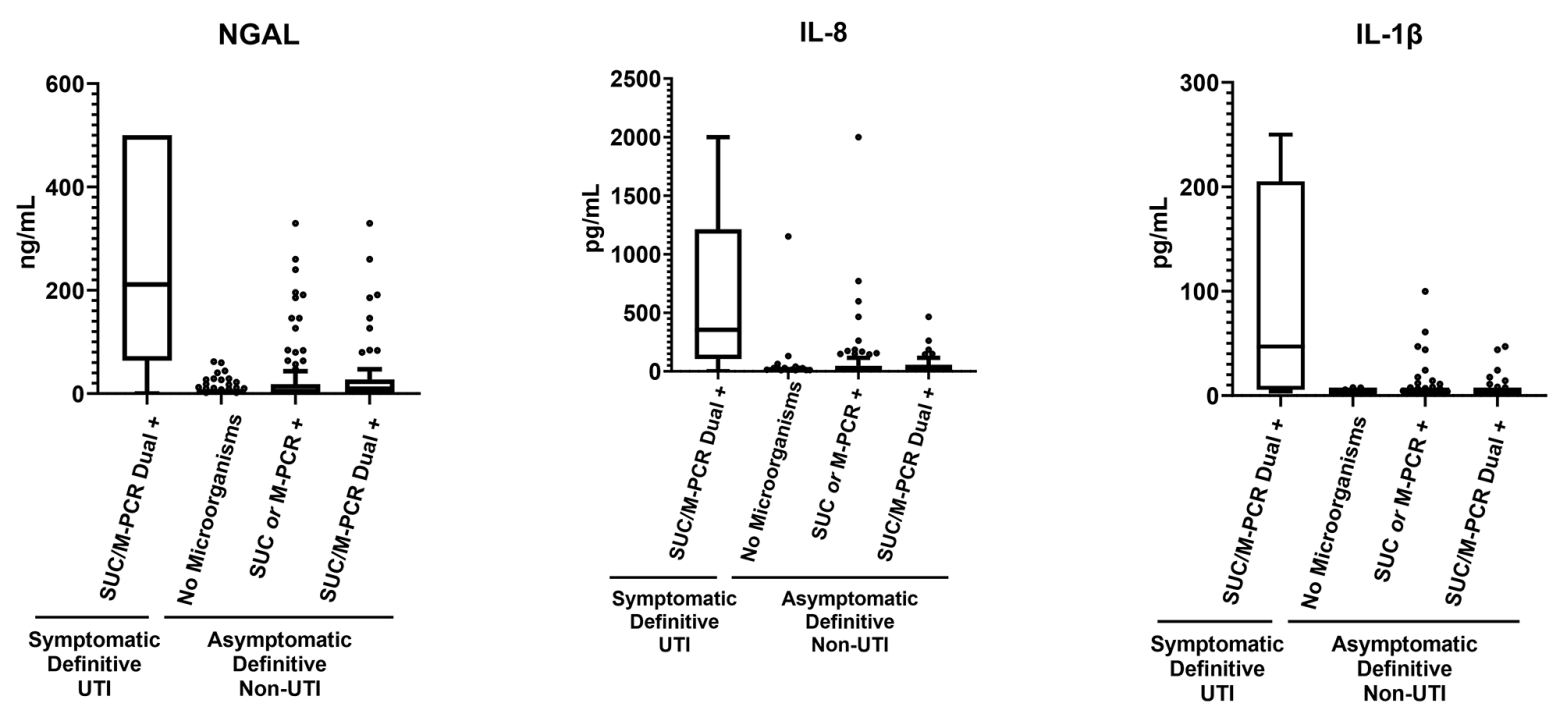
Biomarker Levels are Low in Definitive non-UTIs Regardless of Microbial Detection. Tukey boxplots extending to the 1st and 3rd quartiles with a line at the median indicate the distribution of biomarker (NGAL, IL-8, and IL-1β) levels among each group presented on the x-axis. Biomarker measurements are plotted along the y-axis with each point representing the measurement for a single urine specimen. Groups presented on the x-axis for comparison include “Definitive UTIs” cases (specimens from symptomatic subjects in which microorganisms are detected by both M-PCR and SUC at ≥ 10,000 cells/mL or CFUs/mL respectively), and “Definitive non-UTI” cases (asymptomatic cohort specimens). The “Definitive non-UTI” cases are further divided by microbial detection category: no microbes, microbes detected by SUC *or* M-PCR, and microbes detected by both SUC *and* M-PCR (Dual +)

Levels of all three biomarkers (NGAL, IL, and IL-1β) are significantly lower (*p* < 0.0001) among all asymptomatic cohort specimens, regardless of the presence of detectable microorganisms (Definitive non-UTIs), compared to the symptomatic cohort specimens with microorganisms detected by both SUC and M-PCR (Definitive UTIs) (Fig. 2; Table 2).

**Table 2:** Descriptive Statistics of Biomarker Values for the Definitive UTI and Definitive non-UTI Cohorts Based on Criterion 1

	Definitive UTI (Symptomatic)		Definitive Non-UTI (Asymptomatic)			
			No Microbes		With Microbes	
	SUC and M-PCR +				SUC or M-PCR +	SUC and M-PCR +
**NGAL** **(ng/mL)**						
*n*	351		110		118	51
Minimum	0.16		0.16		0.16	0.16
25th percentile	64.64		0.16		0.16	0.16
Median	211.09		0.16		0.16	9.51
75th percentile	500		0.16		17.65	27.04
Maximum	500		61.99		329.41	329.41
Mean	251.83		4.22		24.44	36.52
SD	193.96		11.33		56.79	70.24
Standard Error	10.35		1.08		5.23	9.84
Lower 95% CI	231.47		2.08		14.08	16.76
Upper 95% CI	272.19		6.36		34.79	56.27
**IL-8** **(pg/mL)**						
*n*	351		110		118	51
Minimum	0		0		0	0
25th percentile	109.12		0		0.38	0.15
Median	355.32		0.34		10.39	14.58
75th percentile	1206.36		3.07		46.98	52.46
Maximum	2000		1152.75		2000	466.02
Mean	693.47		15.57		61.65	46.53
SD	713.79		110.47		208.6	81.16
Standard Error	38.1		10.53		19.2	11.36
Lower 95% CI	618.54		-5.31		23.62	23.7
Upper 95% CI	768.4		36.44		99.69	69.35
**IL-1β** **(pg/mL)**						
*n*	351		110		118	51
Minimum	3.9		3.9		3.9	3.9
25th percentile	5.67		3.9		3.9	3.9
Median	47.07		3.9		3.9	3.9
75th percentile	204.32		3.9		3.9	3.9
Maximum	250		7.68		99.84	47.09
Mean	93.4		3.98		6.56	6.72
SD	97.81		0.53		11.69	8.8
Standard Error	5.22		0.05		1.08	1.23
Lower 95% CI	83.14		3.88		4.42	4.25
Upper 95% CI	103.67		4.08		8.69	9.2

### Individual or consensus biomarker positivity in definitive UTIs and definitive non-UTIs

We then compared the positivity of the individual biomarkers and combinations of biomarkers against symptomatic Definitive UTI cases and Definitive non-UTIs.

#### Definitive UTI percentage

Of 583 specimens from symptomatic subjects with a UTI diagnosed in a specialty setting, bacterial detection ≥ 10,000 by both M-PCR (reported in cells/mL) and by SUC (reported in CFUs/mL) occurred in 351 specimens. These 351 specimens were considered Definitive UTI cases. The 228 asymptomatic subject specimens were considered Definitive non-UTI cases regardless of microbial detection, resulting in a 3:2 ratio of Definitive UTIs to Definitive non-UTIs. It is worth noting that more than half of the asymptomatic group (53.1%, *n* = 122) had detectable microorganisms in the urine at densities > 10,000 cells/mL by M-PCR or CFUs/mL by SUC (asymptomatic bacteriuria), and 28.9% had microbial detection at densities > 10,000 cells/mL and CFUs/mL by both SUC and M-PCR (*n* = 66) (Supplemental Figure S1, Supplemental Table S18).

#### Individual biomarker positivity in distinguishing definitive UTIs and definitive non-UTIs

NGAL was positive in 82.6% (290/351) of definitive UTI cases and negative in 90.8% (207/228) of Definitive non-UTI cases (Table 3). IL-8 was positive in 91.2% (320/351) of Definitive UTI cases and negative in 76.8% (175/228) of definitive non-UTI cases (Table 4). IL-1β was positive in 69.8% (245/351) of definitive UTI cases and negative in 97.9% (221/228) of Definitive non-UTI cases (Table 5).

**Table 3:** NGAL Positivity Contingency Table for Criterion 1

	Definitive UTI	Definitive non-UTI	Total
NGAL Positive	290 (50.1%)	21 (3.6%)	*311 (53.7%)*
NGAL Negative	61 (10.5%)	207 (35.8%)	*268 (46.3%)*
*Total*	*351 (60.6%)*	*228 (39.4%)*	579 (100%)

**Table 4:** IL-8 Positivity Contingency Table for Criterion 1

	Definitive UTI	Definitive non-UTI	Total
IL-8 Positive	320 (55.3%)	53 (9.1%)	*373 (64.4%)*
IL-8 Negative	31 (5.4%)	175 (30.2%)	*206 (35.6%)*
*Total*	*351 (60.6%)*	*228 (39.4%)*	579 (100%)

**Table 5:** IL-1β Positivity Contingency Table for Criterion 1

	Definitive UTI	Definitive non-UTI	Total
IL-1β Positive	245 (42.3%)	7 (1.2%)	*252 (43.5%)*
IL-1β Negative	106 (18.3%)	221 (38.2%)	*327 (56.5%)*
*Total*	*351 (60.6%)*	*228 (39.4%)*	579 (100%)

A statistical analysis summary of the three biomarkers is listed in Table 6. IL-8 had the highest sensitivity (91.2%) while IL-1β had the highest specificity (96.9%).

**Table 6:** Biomarker performance comparisons in the presence of microorganisms based on Criterion 1

Biomarker Performance Characteristics for Differentiating Definitive UTIs from Definitive non-UTIs			
≥ 10,000 Cells/mL and CFUs/mL	NGAL***	IL-8***	IL-1β***
Sensitivity (95% CI)	82.6% (78.2%, 86.4%)	91.2% (87.7%, 93.9%)	69.8% (64.7%, 74.6%)
Specificity (95% CI)	90.8% (86.3%, 94.2%)	76.8% (70.7%, 82.1%)	96.9% (93.8%, 98.8%)
Positive Predictive Value (95% CI)	93.2% (89.9%, 95.8%)	85.8% (81.8%, 89.2%)	97.2% (94.4%, 98.9%)
Negative Predictive Value (95% CI)	77.2% (71.7%, 82.1%)	85.0% (79.3%, 89.5%)	67.6% (62.2%, 72.6%)
Accuracy (95% CI)	85.8% (82.7%, 88.6%)	85.5% (82.4%, 88.3%)	80.5% (77.0%, 83.6%)
Positive Likelihood Ratio (95% CI)	8.97 (5.95, 13.52)	3.92 (3.09, 4.98)	22.74 (10.93, 47.3)
Negative Likelihood Ratio (95% CI)	0.19 (0.13, 0.29)	0.12 (0.09, 0.15)	0.31 (0.15, 0.65)

*** indicates the Proportion Z-test comparison of sensitivity: *p*-value < 0.0001

c. “Consensus” or “All three biomarker” positivity in distinguishing definitive UTIs and definitive non-UTIs “Consensus” is defined as two or more biomarkers meeting or exceeding their respective positivity thresholds. “All three biomarkers” is defined as all three biomarkers meeting or exceeding their respective positivity thresholds (Table 1). Consensus positivity occurred in 84.0% (295/351) of Definitive UTI cases and consensus negativity occurred in 91.2% (208/228) of Definitive non-UTI cases (Table 7). All three biomarkers were positive in 66.1% (232/351) of Definitive UTI cases and negative in 97.4% (222/228) of Definitive non-UTI cases (Table 8).

**Table 7:** Biomarker Consensus Positivity Contingency Table for Criterion 1

	Definitive UTI	Definitive non-UTI	Total
Consensus Positive	295 (50.9%)	20 (3.4%)	*315 (54.4%)*
Consensus Negative	56 (9.7%)	208 (35.9%)	*264 (45.6%)*
Total	*351 (60.6%)*	*228 (39.4%)*	579 (100%)

**Table 8:** All Three Biomarkers Positivity Contingency Table for Criterion 1

	Definitive UTI	Definitive non-UTI		Total
All Three Positive	232 (40.1%)	6 (1.0%)	*238 (41.1%)*	
Less than Three Positive	119 (20.6%)	222 (38.3%)	*341 (58.9%)*	
Total	*351 (60.6%)*	*228 (39.4%)*	579 (100%)	

A summary of the statistical analysis for the biomarker combinations is listed in Table 9. The consensus criteria of at least two biomarkers meeting or exceeding the positivity threshold performed well in terms of both sensitivity and specificity (84.0% and 91.2%, respectively). Although the combination of all three biomarkers being positive had the highest specificity (97.4%), it had lower sensitivity (66.1%).

**Table 9:** Biomarker “Consensus” and “All three biomarkers” performance comparisons Based on Criterion 1

Definitive UTI versus Definitive non-UTI		
≥ 10,000 Cells/mL and CFUs/mL	“Consensus”***	“All three Biomarkers”***
Sensitivity (95% CI)	84.0% (79.8%, 87.7%)	66.1% (60.9%, 71.0%)
Specificity (95% CI)	91.2% (86.8%, 94.6%)	97.4% (94.4%, 99.0%)
Positive Predictive Value (95% CI)	93.7% (90.4%, 96.1%)	97.5% (94.6%, 99.1%)
Negative Predictive Value (95% CI)	78.8% (73.4%, 83.6%)	65.1% (59.8%, 70.2%)
Accuracy (95% CI)	86.9% (83.8%, 89.5%)	78.4% (74.8%, 81.7%)
Positive Likelihood Ratio (95% CI)	9.58 (6.29, 14.6)	25.12 (11.36, 55.51)
Negative Likelihood Ratio (95% CI)	0.17 (0.11, 0.27)	0.35 (0.16, 0.77)

***indicates the Proportion Z-test comparison of sensitivity: *p*-value < 0.0001

## Discussion

To determine if the three infection-associated biomarkers selected for this study (NGAL, IL-8, and IL-1β) [15, 28–32], are both sensitive and specific indicators for UTIs, their levels were measured in both Definitive UTI cases (symptomatic cases, diagnosed in a Urology/Urogynecology specialty setting, with uropathogens identified above threshold values by both SUC and M-PCR) and in Definitive non-UTI control cases (asymptomatic based on FDA-defined criteria included in a Symptom Score Analysis). The Definitive non-UTI cases included asymptomatic individual with detected microbes (asymptomatic bacteriuria). Previous studies had reported the presence of asymptomatic bacteriuria at lower prevalence and primarily in post-menopausal women (up to 5% of healthy pre-menopausal women, up to 25% of post-menopausal women, and up to 1% of healthy adult males) [6–10]. In this study, more than half of this control group (53.1%, *n* = 121) had had microbial detection at densities ≥ 10,000 cells/mL by either SUC *or* M-PCR, and 28.9% had microbial detection at densities ≥ 10,000 cells/mL by both SUC *and* M-PCR (*n* = 66)(Supplemental Figure S1, Supplemental Table S18). This relatively high prevalence of microorganisms in urine specimens from our asymptomatic cohort underscores the importance of practicing diagnostic stewardship, such as implementing clinical testing only for the indicated population of symptomatic cases of presumed UTI, and the value of having these types of biomarkers [33]. 

In this study of more than 800 subjects, the three biomarkers were significantly elevated in symptomatic subjects with positive microbe identification compared to very low biomarker levels in asymptomatic cases with or without microbe identification. Furthermore, we observed a strong positive correlation (R^2^ > 0.90; *p* < 0.0001) between microbial density and urine biomarker levels of NGAL, IL-8, and IL-1β for symptomatic subjects. Biomarker “Consensus” (two or more positive biomarkers) exhibited high accuracy in distinguishing definitive UTI from definitive non-UTI cases, with sensitivity of 90.2%, specificity of 91.2%, positive predictive value (PPV) of 91.7%, negative predictive value (NPV) of 89.7%, and accuracy of 90.7%.

The biomarkers exhibited excellent specificity (> 75% individually and > 90% for consensus) indicating that urine specimens positive for infection-associated biomarkers are highly likely to be associated with cases of active UTIs. There was also a strong correlation between microbe density and rising positivity levels, with high positivity levels in symptomatic patients appearing even at 10,000 cells/mL and CFU/mL in symptomatic patients. Positivity levels for asymptomatic cases remained low even at 100,000 cells/mL and CFU/mL, though there was some increase observed with rising microbe density.

The high sensitivity and specificity (> 90%) of the “Consensus” biomarker model for UTIs makes it a valuable tool to differentiate true UTI cases from asymptomatic bacteriuria and other false-positive differential diagnoses, and also for establishing an objective “truth” for the comparison of existing and novel diagnostic test accuracy. This is especially important since the current “gold standard” test, SUC, is known to have significant limitations, making it an unreliable source of diagnostic “truth.” Specifically, this study (Supplemental Table S20) and others have illustrated the low sensitivity of SUC for non-*E. coli* organisms and polymicrobial infections [16, 34–37].

The main limitation inherent to the use of biobanked urine specimens in this study was the unavailability of detailed medical history including clinical presentation/symptoms, treatment, and clinical outcome records. Additionally, this study was focused on the population 60 years of age and older, based on their higher risk of adverse events from UTIs, however, this selection may limit the applicability of these findings to younger patients.

The measurement of urinary biomarkers, individually or in combination, may also prove valuable as a supportive tool for the clinical diagnostic workup of suspected UTIs, especially in patients unable to clearly communicate their symptoms, such as pediatric patients and patients with cognitive impairment. Leukocyte esterase (LE) dipstick analysis is often employed in clinics as part of the diagnostic workup for UTI, even though the specificity is usually too low to be useful as an individual test (sensitivity range 72–94%; specificity range 9–59%) [15, 30, 31, 38]. The contrasting high accuracy of the consensus biomarker model detailed here indicates it could be a superior tool for assisting in the diagnosis of UTI.

## Conclusions

Using symptomatic subjects’ urine specimens in which SUC and M-PCR results agreed on the presence of uropathogens, we demonstrated the association of NGAL, IL-8, and IL-1β, with Definitive UTI cases. A consensus criterion with ≥ 2 of the biomarkers meeting the positivity thresholds showed a good balance of sensitivity (84.0%), specificity (91.2%), and accuracy (86.9%), making it an excellent supportive diagnostic tool for resolving the presence of active UTI, particularly if SUC and M-PCR results disagree. These biomarkers can be used as an important supplemental tool to determine if a case is a UTI when the microbial detection and identification diagnostic test has significant limitations in sensitivity or when it is unclear whether the detected microorganism(s) are causing disease.

### Electronic supplementary material

Below is the link to the electronic supplementary material.

Supplementary Material 1

## Data Availability

The data presented in this study are available on request from the corresponding author. The data are not publicly available due to privacy concerns.

## Abbreviations

ACSS: American English Acute Cystitis Symptom Score
CNA/MAC: Colistin and nalidixic acid agar/MacConkey agar
CoNS: Coagulase negative *staphylococci* group
ELISA: Enzyme-linked immunosorbent assay
EQUC: Enhanced-quantitative urine culture
IL: Interleukin
M-PCR: Multiplex polymerase chain reaction
NGAL: Neutrophil gelatinase-associated lipocalin (NGAL)
NPV: Negative predictive value
P-AST: Pooled antibiotic susceptibility test
PPV: Positive predictive value
SUC: Standard urine culture
UTI: Urinary tract infection
VGS: Viridans group *streptococci*
