# Supplementary material for: Urine biomarkers individually and as a consensus model show high sensitivity and specificity for detecting UTIs
Source: BMC Infect Dis. 2024 Jan 31;24:153. doi: 10.1186/s12879-024-09044-2 (PMC10829179; doi:10.1186/s12879-024-09044-2)

Supplemental Materials

Urine Biomarkers Individually and as a Consensus Model Show High Sensitivity and Specificity for Detecting UTIs

Marzieh Akhlaghpour^1^, Emery Haley^2^, Laura Parnell^3^, Natalie Luke^2^, Mohit Mathur^4^, Richard Festa^1^, Michael Percaccio^1^, Jesus Magallon^1^, Mariana Remedios-Chan^1^, Alain Rosas^1^, Jimin Wang^5^, Yan Jiang^5^, Lori Anderson^6^, David Baunoch^1*^

^1^Department of Research and Development, Pathnostics, 15545 Sand Canyon Suite 100, Irvine, CA, USA 92618

^2^Department of Clinical Research, Pathnostics, 15545 Sand Canyon Suite 100, Irvine, CA, USA 92618

^3^Department of Scientific Writing, Precision Consulting, 6522 Harbor Mist, Missouri City, TX, USA 77459

^4^Department of Medical Affairs, Pathnostics, 15545 Sand Canyon Suite 100, Irvine, CA, USA 92618

^5^Department of Statistical Analysis, Stat4Ward, 2 Edgemoor Lane, Pittsburgh, PA, USA 15238

^6^Department of Diagnostic Market Access, Pathnostics, 15545 Sand Canyon Suite 100, Irvine, CA, USA 92618

*****Correspondence: dbaunoch@pathnostics.com; Tel.: 714-966-1221.

1. **Supplemental Table S1**. ICD-10-CM codes for the symptomatic cohort.

| **ICD-10-CM Code** | **Code Description** | **Frequency** | **Percent** |
| --- | --- | --- | --- |
| N39.0 | Urinary tract infection, site not specified | 534 | 81.8 |
| R30.0 | Dysuria | 43 | 6.6 |
| R31.0 | Gross hematuria | 25 | 3.8 |
| Z87.440 | Personal history of diseases of urinary system | 5 | 0.8 |
| R31.9 | Hematuria, unspecified | 4 | 0.6 |
| R82.998 | Other abnormal findings in urine | 4 | 0.6 |
| Others | - | 38 | 12.4 |

Some patients had more than one ICD-10-CM code associated with their case. The five most prevalent codes are listed individually, and all remaining codes were grouped together as “other”.

1. **Supplemental Table S2**. Sensitivity and specificity of candidate biomarkers in the pilot study (n = 100).

|  | **MMP-9** | **NGAL** | **IL-8** | **IL-6** | **IL-1β** |
| --- | --- | --- | --- | --- | --- |
| **Sensitivity** | 70.59 | 85.29 | 100 | 50 | 85.29 |
| **Specificity** | 85.19 | 85.19 | 51.85 | 70.37 | 85.19 |

1. Addition results not included in the main manuscript based on Criterion 1, Biomarker Positivity Performance Using Microbial Density Detection ≥ 10, 000 cells/mL by M-PCR and CFUs/mL by SUC.

*Biomarker and microbial comparison defined by combinations of two biomarkers (NGAL & IL-8, NGAL & IL-1β, IL-8 & IL-1β)* – NGAL and IL-8 were positive in 80.3% (282/351) of Definitive UTIs and negative in 91.7% (209/228) of Definitive Non-UTIs (Supplemental Table S3). NGAL and IL-1β were positive in 66.4% (233/351) of Definitive UTIs and negative in 96.9% (221/228) of Definitive non-UTIs (Supplemental Table S4). IL-8 and IL-1β was positive in 69.5% (244/351) of Definitive UTIs and negative in 97.4% (222/228) of Definitive non-UTIs (Supplemental Table S5).

**Supplemental Table S3**. NGAL and IL-8 Positivity Contingency Table

|  | **Definitive UTI** | **Definitive Non-UTI** | *Total* |
| --- | --- | --- | --- |
| **NGAL and IL-8 Positive** | 282 (48.7%) | 19 (3.3%) | *301 (52.0%)* |
| **NGAL and IL-8 Negative** | 69 (11.9%) | 209 (36.1%) | *278 (48.0%)* |
| *Total* | *351 (60.6%)* | *228 (39.4%)* | 579 (100%) |

**Supplemental Table S4**. NGAL and IL-1β Positivity Contingency Table

|  | **Definitive UTI** | **Definitive Non-UTI** | *Total* |
| --- | --- | --- | --- |
| **NGAL and IL-1β Positive** | 233 (40.2%) | 7 (1.2%) | *240 (41.5%)* |
| **NGAL and IL-1β Negative** | 118 (20.4%) | 221 (38.2%) | *339 (58.5%)* |
| *Total* | *351 (60.6%)* | *228 (39.4%)* | 579 (100%) |

**Supplemental Table S5**. IL-8 and IL-1β Positivity Contingency Table

|  | **Definitive UTI** | **Definitive Non-UTI** | *Total* |
| --- | --- | --- | --- |
| **IL-8 and IL-1β Positive** | 244 (42.1%) | 6 (1.0%) | *250 (43.2%)* |
| **IL-8 and IL-1β Negative** | 107 (18.5%) | 222 (38.3%) | *329 (56.8%)* |
| *Total* | *351 (60.6%)* | *228 (39.4%)* | 579 (100%) |

*A statistical analysis summary of the three combinations of biomarkers* is listed in Supplemental Table S6. The sensitivities of the dual combinations of biomarkers were statistically significantly different (p < 0.0001).

**Supplemental Table S6**. Combination of two biomarkers performance comparisons in the presence of microorganisms detected at both ≥ 10^4^ cells/mL by M-PCR and ≥ 10^4^ CFUs/mL by SUC.

| Definitive UTI versus Definitive Non-UTI | | | |
| --- | --- | --- | --- |
| ≥ 10,000 Cells/mL and CFUs/mL | **NGAL & IL-8***** | **NGAL & IL-1β***** | **IL-8 & IL-1β***** |
| Sensitivity (95% CI) | 80.3% (75.8%, 84.4%) | 66.4% (61.2%, 71.3%) | 69.5% (64.4%, 74.3%) |
| Specificity (95% CI) | 91.7% (87.3%, 94.9%) | 96.9% (93.8%, 98.8%) | 97.4% (94.4%, 99.0%) |
| Positive Predictive Value (95% CI) | 93.7% (90.3%, 96.2%) | 97.1% (94.1%, 98.8%) | 97.6% (94.8%, 99.1%) |
| Negative Predictive Value (95% CI) | 75.2% (69.7%, 80.1%) | 65.2% (59.9%, 70.3%) | 67.5% (62.1%, 72.5%) |
| Accuracy | 84.8% (81.6%, 87.6%) | 78.4% (74.8%, 81.7%) | 80.5% (77.0%, 83.6%) |
| Definitive UTI Percentage | 60.6% | 60.6% | 60.6% |
| Positive Likelihood Ratio | 9.64 (6.25, 14.87) | 21.62 (10.39, 45.01) | 26.42 (11.96, 58.36) |
| Negative Likelihood Ratio | 0.21 (0.14, 0.33) | 0.35 (0.17, 0.72) | 0.31 (0.14, 0.69) |

***The Proportion Z-test comparison of sensitivity: *p*-value < 0.0001

IV. Results based on criterion 2, Biomarker Positivity Performance Using Microbial Density Detection ≥ 100, 000 cells/mL by M-PCR and CFUs/mL by SUC.

1. Definitive UTI Percentage

Out of the 583 symptomatic subject specimens, bacterial detection ≥ 10,000 cells/mL by M-PCR and CFUs/mL by SUC occurred in 245 specimens. These 245 specimens were considered Definitive UTIs. The 228 asymptomatic subject specimens were considered Definitive non-UTIs regardless of microbial detection. Therefore, the Definitive UTI percentage defined by criterion 2 was 51.8% ((245/(245+228))*100).

1. Biomarker and microbial comparison defined by each biomarker

NGAL was positive (≥ 38 ng/mL) in 89.0% (218/245) of Definitive UTIs and negative in 90.8% (207/228) of Definitive non-UTIs (Supplemental Table S7). IL-8 was positive (≥ 20.6 pg/mL) in 94.3% (231/245) of Definitive UTIs and negative in 76.8% (175/228) of Definitive non-UTIs (Supplemental Table S8). IL-1β was positive (≥ 12.4 pg/mL) in 75.5% (185/245) of Definitive UTIs and negative in 96.9% (221/228) of Definitive non-UTIs (Supplemental Table S9).

**Supplemental Table S7**. NGAL Positivity Contingency Table for Criterion 2

|  | **Definitive UTI** | **Definitive Non-UTI** | *Total* |
| --- | --- | --- | --- |
| **NGAL Positive** | 218 (46.1%) | 21 (4.4%) | *239 (50.5%)* |
| **NGAL Negative** | 27 (5.7%) | 207 (43.8%) | *234 (49.5%)* |
| *Total* | *245 (51.8%)* | *228 (48.2%)* | 473 (100%) |

**Supplemental Table S8**. IL-8 Positivity Contingency Table for Criterion 2

|  | **Definitive UTI** | **Definitive Non-UTI** | *Total* |
| --- | --- | --- | --- |
| **IL-8 Positive** | 231 (48.8%) | 53 (11.2%) | *284 (60.0%)* |
| **IL-8 Negative** | 14 (3.0%) | 175 (37.0%) | *189 (40.0%)* |
| *Total* | *245 (51.8%)* | *228 (48.2%)* | 473 (100%) |

**Supplemental Table S9**. IL-1β Positivity Contingency Table for Criterion 2

|  | **Definitive UTI** | **Definitive Non-UTI** | *Total* |
| --- | --- | --- | --- |
| **IL-1β Positive** | 185 (39.1%) | 7 (1.5%) | *192 (40.6%)* |
| **IL-1β Negative** | 60 (12.6%) | 221 (46.7%) | *281 (59.4%)* |
| *Total* | *245 (51.8%)* | *228 (48.2%)* | 473 (100%) |

*A statistical analysis summary of the three biomarkers* is listed in Supplemental Table S10. The sensitivity of each biomarker was statistically different (p < 0.0001). IL-8 had the highest sensitivity (94.3%) while IL-1β had the highest specificity (96.9%).

**Supplemental Table S10**. Biomarker performance comparisons in the presence of microorganisms detected at both ≥ 10^5^ cells/mL by M-PCR and ≥ 10^5^ CFUs/mL by SUC (criterion 2).

| Definitive UTI versus Definitive Non-UTI | | | |
| --- | --- | --- | --- |
| ≥ 10^5^ Cells/mL and CFUs/mL | **NGAL***** | **IL-8***** | **IL-1β***** |
| Sensitivity (95% CI) | 89.0% (84.4%, 92.6%) | 94.3% (90.6%, 96.8%) | 75.5% (69.6%, 80.8%) |
| Specificity (95% CI) | 90.8% (86.3%, 94.2%) | 76.8% (70.7%, 82.1%) | 96.9% (93.8%, 98.8%) |
| Positive Predictive Value (95% CI) | 91.2% (86.9%, 94.5%) | 81.3% (76.3%, 85.7%) | 96.4% (92.6%, 98.5%) |
| Negative Predictive Value (95% CI) | 88.5% (83.7%, 92.3%) | 92.6% (87.9%, 95.9%) | 78.6% (73.4%, 83.3%) |
| Accuracy | 89.9% (86.8%, 92.4%) | 85.8% (82.4%, 88.9%) | 85.8% (82.4%, 88.9%) |
| Positive Likelihood Ratio | 9.66 (6.41, 14.56) | 4.06 (3.2, 5.15) | 24.59 (11.82, 51.18) |
| Negative Likelihood Ratio | 0.12 (0.08, 0.18) | 0.07 (0.06, 0.09) | 0.25 (0.12, 0.53) |

***The Proportion Z-test comparison of sensitivity: *p*-value < 0.0001

1. *Biomarker consensus results based on criterion 2:* “Consensus” is defined as > 2 biomarkers meeting or exceeding the positivity threshold. Consensus positivity occurred in 90.2% (221/245) of Definitive UTIs and consensus negativity occurred in 91.2% (208/228) of Definitive non-UTIs (Supplemental Table S11). All three biomarkers were positive in 72.7% (178/245) of Definitive UTIs and negative in 97.4% (222/228) of Definitive non-UTIs (Supplemental Table S12).

**Supplemental Table S11**. Biomarker Consensus Positivity Contingency Table for Criterion 2

|  | **Definitive UTI** | **Definitive Non-UTI** | *Total* |
| --- | --- | --- | --- |
| **Consensus Positive** | 221 (46.7%) | 20 (4.2%) | *241 (51.0%)* |
| **Consensus Negative** | 24 (5.1%) | 208 (44.0%) | *232 (49.0%)* |
| *Total* | *245 (51.8%)* | *228 (48.2%)* | 473 (100%) |

**Supplemental Table S12**. All Three Biomarkers Positivity Contingency Table for Criterion 2

|  | **Definitive UTI** | **Definitive Non-UTI** | *Total* |
| --- | --- | --- | --- |
| **All Three Positive** | 178 (37.6%) | 6 (1.3%) | *184 (38.9%)* |
| **Less Than Three Positive** | 67 (14.2%) | 222 (46.9%) | *289 (61.1%)* |
| *Total* | *245 (51.8%)* | *228 (48.2%)* | 473 (100%) |

A statistical analysis summary of the biomarker combinations is listed in Supplemental Table S13. The sensitivity of each biomarker combination was statistically significantly different (p < 0.0001). Although the combination of all three biomarkers being positive had the highest specificity (97.4%), it had relatively low sensitivity (72.7%). The consensus criteria with at least 2 biomarkers meeting or exceeding the positivity threshold performed well in terms of both sensitivity and specificity (90.2% and 91.2%, respectively).

**Supplemental Table S13**. Biomarker “Consensus” and triple combination performance comparisons in the presence of microorganisms detected at both ≥ 10^5^ cells/mL by M-PCR and CFUs/mL by SUC (criterion 2).

| Definitive UTI versus Definitive Non-UTI | | |
| --- | --- | --- |
| ≥ 10^5^ Cells/mL and CFUs/mL | **“Consensus”***** | **“All three Biomarkers”***** |
| Sensitivity (95% CI) | 90.2% (85.8%, 93.6%) | 72.7% (66.6%, 78.1%) |
| Specificity (95% CI) | 91.2% (86.8%, 94.6%) | 97.4% (94.4%, 99.0%) |
| Positive Predictive Value (95% CI) | 91.7% (87.5%, 94.9%) | 96.7% (93.0%, 98.8%) |
| Negative Predictive Value (95% CI) | 89.7% (85.0%, 93.3%) | 76.8% (71.5%, 81.6%) |
| Accuracy | 90.7% (87.7%, 93.2%) | 84.6% (81.0%, 87.7%) |
|  |  |  |
| Positive Likelihood Ratio | 10.28 (6.75, 15.66) | 27.61 (12.49, 61.03) |
| Negative Likelihood Ratio | 0.11 (0.07, 0.16) | 0.28 (0.13, 0.62) |

***The Proportion Z-test comparison of sensitivity: p-value < 0.0001

1. *Biomarker and microbial comparison defined by combinations of two biomarkers (NGAL & IL-8, NGAL & IL-1β, IL-8 & IL-1β)*- NGAL and IL-8 were positive in 87.3% (213/245) of Definitive UTIs and negative in 91.7% (209/228) of Definitive non-UTIs (Supplemental Table S14). NGAL and IL-1β were positive in 73.1% (179/245) of Definitive UTIs and negative in 96.9% (221/228) of Definitive non-UTIs (Supplemental Table S15). IL-8 and IL-1β were positive in 75.1% (184/245) of Definitive UTIs and negative in 97.4% (222/228) of Definitive non-UTIs (Supplemental Table S16).

**Supplemental Table S14**. NGAL and IL-8 Positivity Contingency Table (Criterion 2)

|  | **Definitive UTI** | **Definitive Non-UTI** | *Total* |
| --- | --- | --- | --- |
| **NGAL and IL-8 Positive** | 214 (45.2%) | 19 (4.0%) | *233 (49.3%)* |
| **NGAL and IL-8 Negative** | 31 (6.6%) | 209 (44.2%) | *240 (50.7%)* |
| *Total* | *245 (51.8%)* | *228 (48.2%)* | 473 (100%) |

**Supplemental Table S15**. NGAL and IL-1β Positivity Contingency Table (Criterion 2)

|  | **Definitive UTI** | **Definitive Non-UTI** | *Total* |
| --- | --- | --- | --- |
| **NGAL and IL-1β Positive** | 179 (37.8%) | 7 (1.5%) | *186 (39.3%)* |
| **NGAL and IL-1β Negative** | 66 (14.0%) | 221 (46.7%) | *287 (60.7%)* |
| *Total* | *245 (51.8%)* | *228 (48.2%)* | 473 (100%) |

**Supplemental Table S16**. IL-8 and IL-1β Positivity Contingency Table (Criterion 2)

|  | **Definitive UTI** | **Definitive Non-UTI** | *Total* |
| --- | --- | --- | --- |
| IL-8 and IL-1β Positive | 184 (38.9%) | 6 (1.3%) | *190 (40.2%)* |
| IL-8 and IL-1β Negative | 61 (12.9%) | 222 (46.9%) | *283 (59.8%)* |
| *Total* | *245 (51.8%)* | *228 (48.2%)* | 473 (100%) |

A statistical analysis summary of the three combinations of biomarkers is listed in Supplemental Table S17. The sensitivities of the dual combinations of biomarkers were statistically significantly different (p = 0.00017).

**Supplemental Table S17**. Biomarker combination performance comparisons in the presence of microorganisms detected at both ≥ 10^5^ cells/mL by M-PCR and CFUs/mL by SUC (Criterion 2).

| Definitive UTI versus Definitive Non-UTI | | | |
| --- | --- | --- | --- |
| ≥ 10^5^ Cells/mL and CFUs/mL | **NGAL & IL-8** | **NGAL & IL-1β** | **IL-8 & IL-1β** |
| Sensitivity (95% CI) | 87.3% (82.5%, 91.2%) | 73.1% (67.0%, 78.5%) | 75.1% (69.2%, 80.4%) |
| Specificity (95% CI) | 91.7% (87.3%, 94.9%) | 96.9% (93.8%, 98.8%) | 97.4% (94.4%, 99.0%) |
| Positive Predictive Value (95% CI) | 91.8% (87.6%, 95.0%) | 96.2% (92.4%, 98.5%) | 96.8% (93.3%, 98.8%) |
| Negative Predictive Value (95% CI) | 87.1% (82.2%, 91.1%) | 77.0% (71.7%, 81.7%) | 78.4% (73.2%, 83.1%) |
| Accuracy | 89.4% (86.3%, 92.1%) | 84.6% (81.0%, 87.7%) | 85.8% (82.4%, 88.9%) |
| Positive Likelihood Ratio | 10.48 (6.8, 16.16) | 23.8 (11.43, 49.54) | 28.54 (12.92, 63.06) |
| Negative Likelihood Ratio | 0.14 (0.09, 0.21) | 0.28 (0.13, 0.58) | 0.26 (0.12, 0.57) |

***The Proportion Z-test comparison of sensitivity:  p-value <0.0001

**V. Supplemental Figure S1.** Asymptomatic Microbial Density. The bacterial density for each asymptomatic case as measured by M-PCR, with the median density (red line), at 239,611 and the mean density of 2.8 x 10^7^ Cells/mL. Each individual dot within the figure represents a distinct specimen, highlighting the diverse range of densities observed within the dataset. This figure visually captures the variability and trends in bacterial densities within asymptomatic bacteriuria cases.

**VI. Supplemental Table S18**. Asymptomatic Subjects with Positive Microbial Detection (> 10,000 CFU or cells/mL for bacteria/bacterial groups and any cell density for yeasts) by SUC and/or M-PCR.

| **n = 228** | **SUC +** | **M-PCR +** | **SUC or M-PCR +** | **SUC and -PCR +** |  |
| --- | --- | --- | --- | --- | --- |
|  |  |  |  |  |  |
| **Male (n = 101)** | 16 | 21 | 24 | 13 |  |
| **Female (n = 127)** | 56 | 94 | 97 | 53 |  |
| **Total (n, %)** | 72, 31.6% | 115, 50.4% | 121, 53.1% | 66, 28.9% |  |

Supplemental Table S19. Medical History Questions for Asymptomatic Volunteers

| Question | Answer Options |
| --- | --- |
| In the last 12 months, how many times have you been seen or treated for a UTI? | - 0 - 1 - 2 - >3 - Don’t know |
| Are you currently taking oral steroids? | - Yes - No - Don’t know |
| Are you currently being treated for a UTI? | - Yes - No - Don’t know |
| Have you ever had reconstructive surgery performed on your bladder? | - Yes - No - Don’t know |
| Have you had renal stone(s) in the past two years? | - Yes - No - Don’t know |
| Were you diagnosed with bladder cancer within the last three years? | - Yes - No - Don’t know |
| Do you have a transplanted organ? | - Yes - No - Don’t know |
| Have you been diagnosed with an autoimmune disease or are you currently taking drugs to suppress your immune system? | - Yes - No - Don’t know |
| Are you diabetic? | - No - Yes, Type I - Yes, Type II - Don’t know |
| Are you currently taking antibiotics? | - Yes - No - Don’t know   If yes, what is the name of the antibiotic? ___________________________ |

Supplemental Table S20. Organisms Detection Frequency by SUC and M-PCR in Symptomatic and Asymptomatic Cohorts.


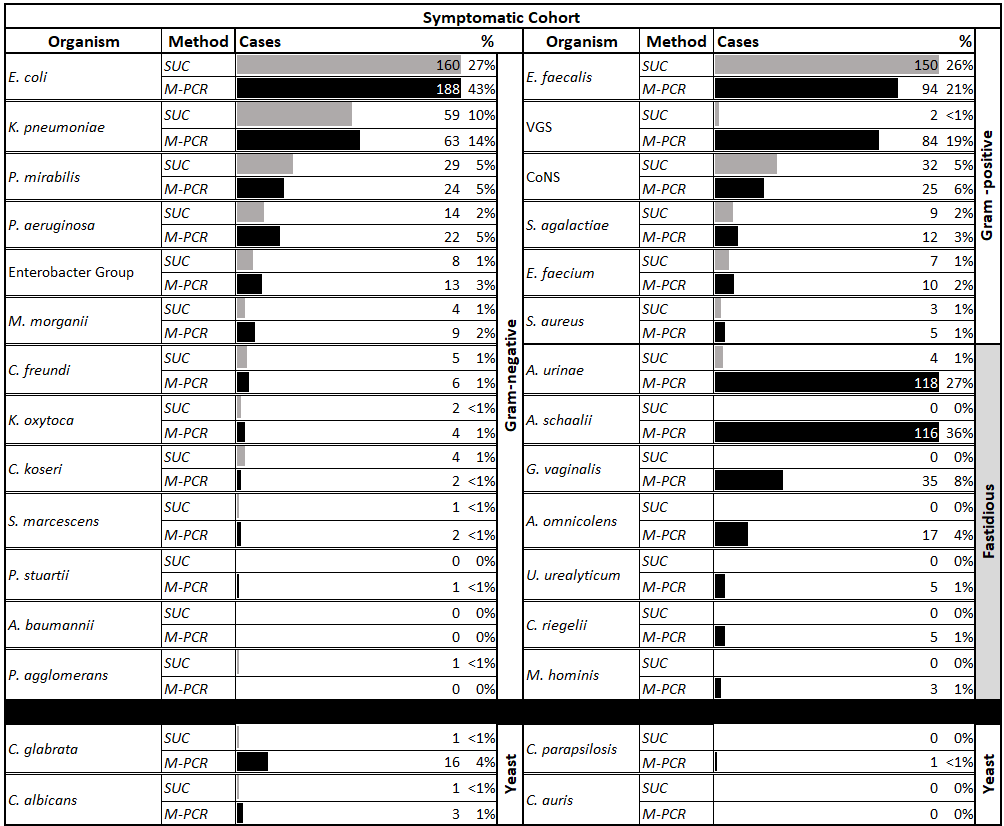


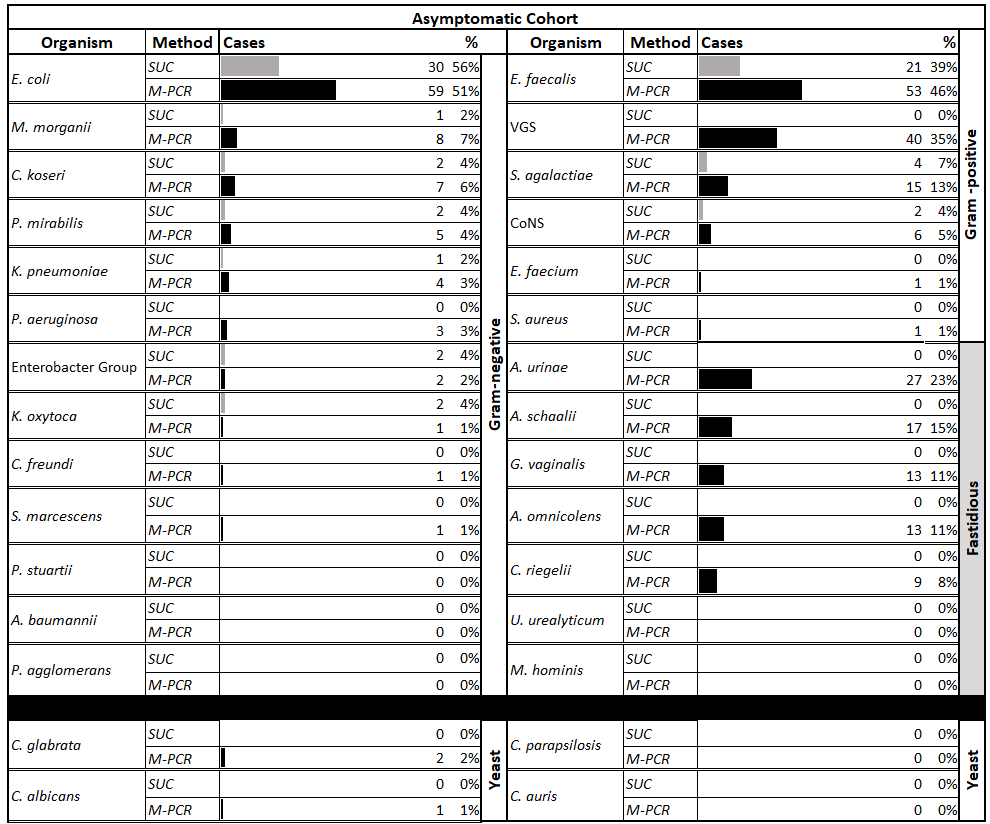

Supplement: Supplementary file 1 — Supplementary Material 1 [file 12879_2024_9044_MOESM1_ESM.docx]
